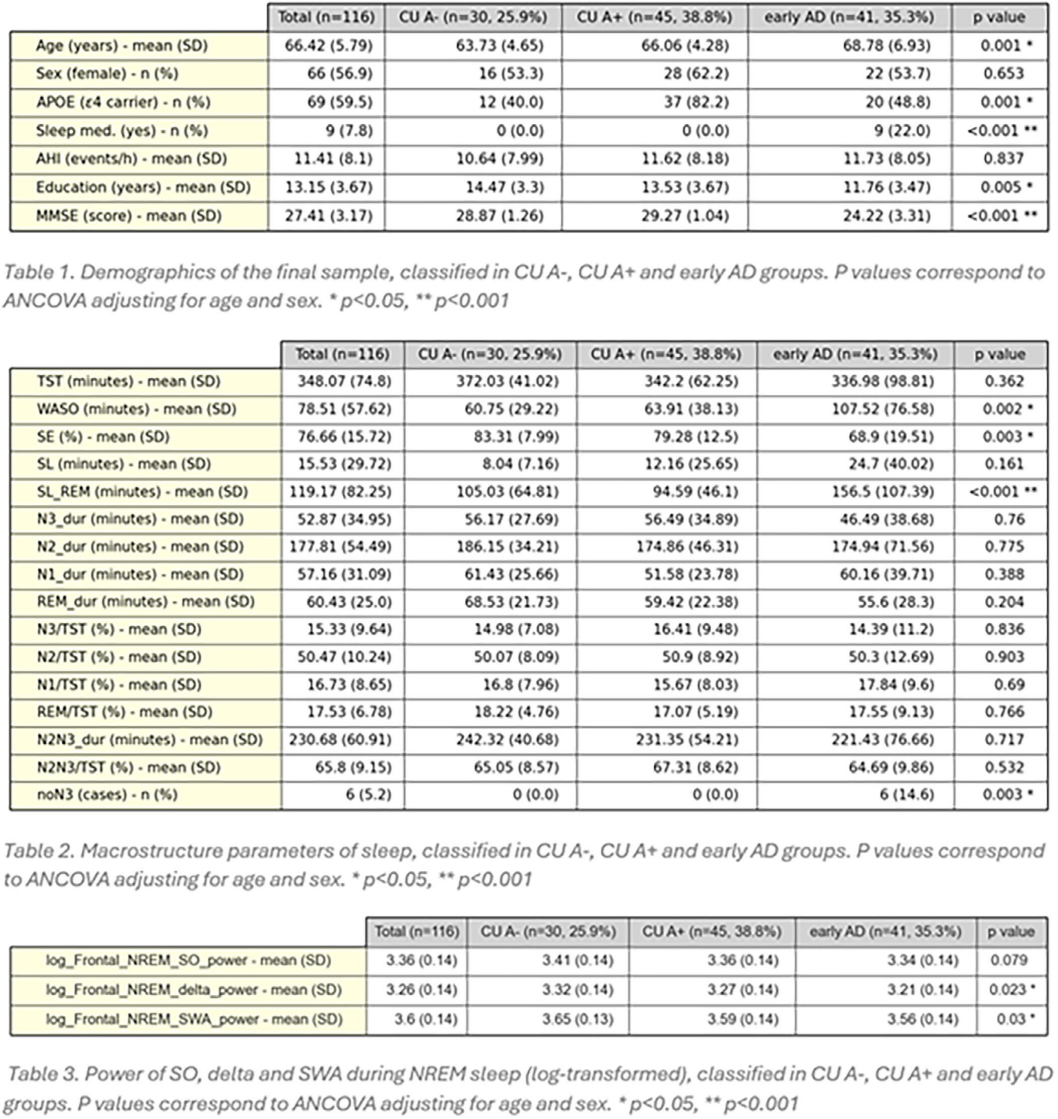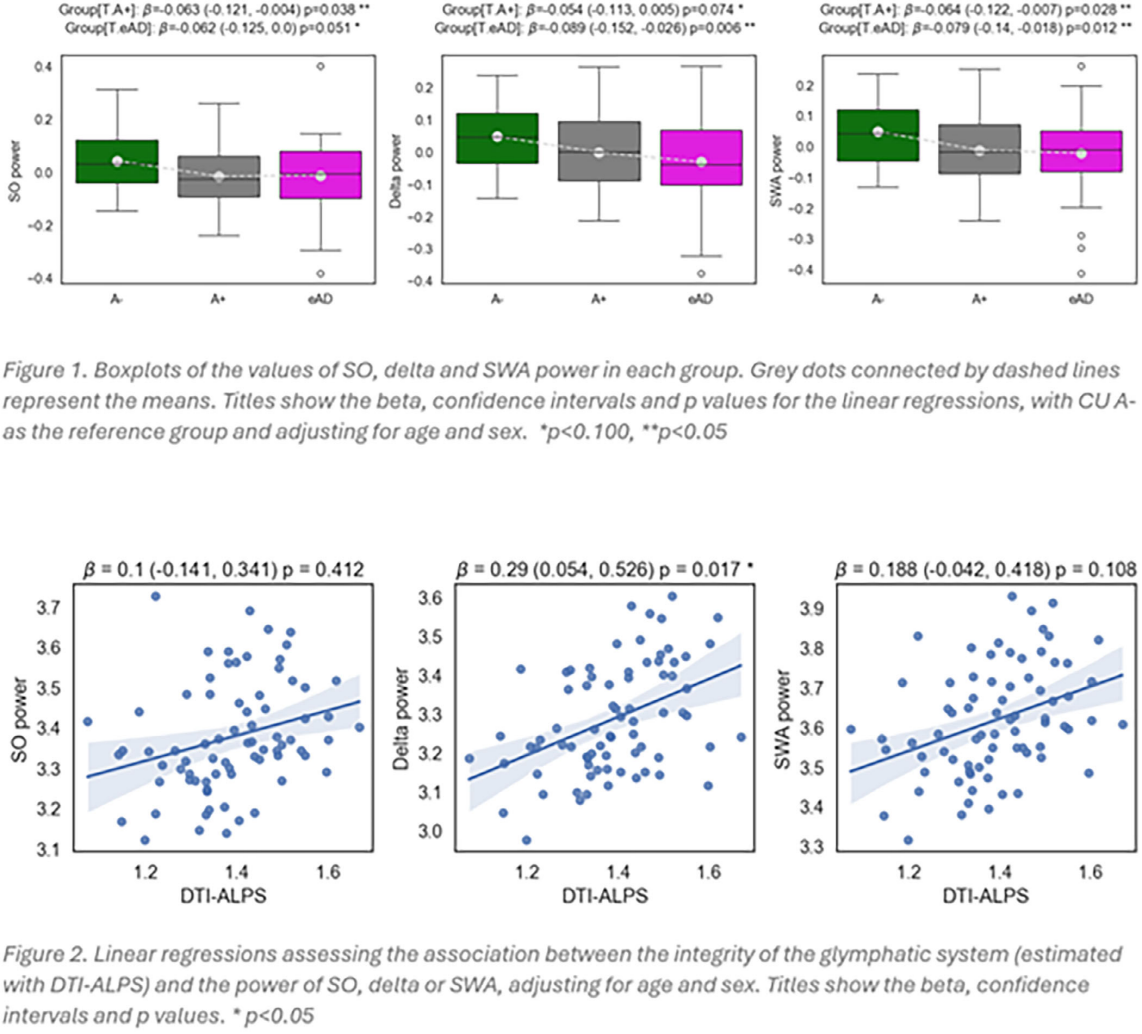# Reduction of slow wave activity during deep sleep in the Alzheimer's disease continuum

**DOI:** 10.1002/alz70855_099178

**Published:** 2025-12-23

**Authors:** Núria Tort‐Colet

**Affiliations:** ^1^ Barcelonaβeta Brain Research Center (BBRC), Pasqual Maragall Foundation, Barcelona, Spain; Hospital del Mar Research Institute, Barcelona, Spain

## Abstract

**Background:**

Many Alzheimer's disease (AD) patients experience sleep disturbances and slow wave activity (SWA) alterations have been linked to tau and amyloid (A) β pathology, possibly through glymphatic system (GS) disturbance. Here, we evaluate SWA and GS's integrity at different AD continuum stages: from cognitively unimpaired (CU) A negative (A‐) participants to early AD (eAD) patients.

**Methods:**

90 CU ALFAsleep participants (65 years, 51% females, 67% APOE‐ε4 carriers), and 58 eAD patients from the Clínic Alzheimer's Disease Sleep Cohort from Hospital Clinic de Barcelona (HCB) (69 years, 50% females, 43.1% APOE‐ε4carriers, 36 MCI and 22 mild AD dementia) underwent video‐polysomnography (PSG) in the HCB Sleep Unit. 33.9% CU were classified as A positive (A+) based on either CSF Aβ42/40 ratio (<0.071, Roche NeuroToolKit, available in 81 CU) or Aβ [^18^F] (flutemetamol) PET (>12 Centiloids, available in 66 CU). eAD patients were diagnosed by a panel of neurologists and neuropsychologists and had a biomarker profile consistent with AD (54 based on CSF, 4 on PET). Participants with apnea‐hypopnea index > 30 were excluded. The final sample consisted of 116 participants: 30 (CU) A‐, 45 (CU) A+ and 41 eAD. We computed the averaged power at the slow oscillation (SO, 0.5‐1Hz), delta (1‐4Hz) and SWA (0.5‐4Hz) EEG frequency bands from non‐rapid eye movement (NREM) sleep stages N2 and N3 at frontal electrodes (F3 and F4). Diffusion tensor image analysis along the perivascular space (DTI‐ALPS, Takoaet al. 2017) was used to assess perivascular fluid dynamics, providing an indirect estimate of GS function in CU participants. We used age and sex adjusted ANCOVA and linear regressions to evaluate differences in sleep across the AD continuum, as well as associations with DTI‐ALPS.

**Results:**

Compared to other groups, eAD had higher wake after sleep onset (*p* = 0.002) and REM latency (*p* <0.001), and lower sleep efficiency (*p* = 0.003). Moreover, compared to A‐, A+ and eAD had lower SO (*p* = 0.038 and *p* = 0.051), delta (*p* = 0.074 and *p* = 0.006), and SWA (*p* = 0.028 and *p* = 0.012) power. Among CU, DTI‐ALPS was positively associated with delta and SWA power (*p* = 0.017, *p* = 0.108).

**Conclusion:**

AD progression is associated with SWA alterations which might promote Aβ accumulation through the GS impairment.